# Comparative studies between humans and golden Syrian hamsters via thromboelastography

**DOI:** 10.1002/ame2.12403

**Published:** 2024-05-20

**Authors:** Ze Yang, Lili Xie, Jingjing Ba, Simin Zan, Letong Zhang, Xinyi Zhang, Yang Yu

**Affiliations:** ^1^ School of Laboratory Animal & Shandong Laboratory Animal Center Shandong First Medical University & Shandong Academy of Medical Sciences Jinan China; ^2^ The Second Affiliated Hospital of Shandong First Medical University Taian China

**Keywords:** platelet function, Syrian hamster, thromboelastography (TEG), thrombosis

## Abstract

**Background:**

Thromboelastography (TEG) is a widely utilized clinical testing method for real‐time monitoring of platelet function and the thrombosis process. Lipid metabolism disorders are crucial risk factors for thrombosis. The lipid metabolism characteristics of hamsters resemble those of humans more closely than mice and rats, and their relatively large blood volume makes them suitable for studying the mechanisms of thrombosis related to plasma lipid mechanisms. Whole blood samples from golden Syrian hamsters and healthy humans were obtained following standard clinical procedures. TEG was employed to evaluate coagulation factor function, fibrinogen (Fib) function, platelet function, and the fibrinolytic system.

**Methods:**

The whole blood from hamster or healthy human was isolated following the clinical procedure, and TEG was employed to evaluate the coagulation factor function, Fib function, platelet function, and fibrinolytic system. Coagulation analysis used ACLTOP750 automatic coagulation analysis pipeline. Blood routine testing used XN‐2000 automatic blood analyzer.

**Results:**

TEG parameters revealed that hamsters exhibited stronger coagulation factor function than humans (reaction time [R], *p* = 0.0117), with stronger Fib function (alpha angle, *p* < 0.0001; K‐time [K], *p* < 0.0001). Platelet function did not differ significantly (maximum amplitude [MA], *p* = 0.077). Hamsters displayed higher coagulation status than humans (coagulation index [CI], *p* = 0.0023), and the rate of blood clot dissolution in hamsters differed from that in humans (percentage lysis 30 min after MA, *p* = 0.02). Coagulation analysis parameters indicated that prothrombin time (PT) and activated partial thromboplastin time (APTT) were faster in hamsters than in humans (PT, *p* = 0.0014; APTT, *p* = 0.03), whereas the Fib content was significantly lower in hamsters than in humans (*p* < 0.0001). No significant difference was observed in thrombin time (*p* = 0.1949).

**Conclusions:**

In summary, TEG could be used to evaluate thrombosis and bleeding parameters in whole blood samples from hamsters. The platelet function of hamsters closely resembled that of humans, whereas their coagulation function was significantly stronger.

## 1 INTRODUCTION

Cardiovascular diseases, primarily caused by atherosclerotic plaque and thrombosis, are the leading cause of death. These conditions are closely associated with lipid metabolism disorders.^1^ When the plaque ruptures, platelets are activated by exposed collagen, triggering a cascade reaction in the coagulation process, further leading to thrombosis formation and arterial blood flow obstruction, resulting in acute cardiovascular events with potentially life‐threatening consequences.^2^ Consequently, it is crucial to study these cardiovascular diseases using appropriate animal models and monitoring methods.

Small rodents are commonly employed to investigate the mechanisms of thrombosis resulting from lipid metabolism disorders. Golden Syrian hamsters (*Mesocricetus auratus*), referred to as hamsters in this study, possess lipoprotein metabolism characteristics similar to humans,^3^ such as ceramide (cer) and phosphatidyl inositol (PI), which are often utilized to identify metabolic abnormalities of early atherosclerosis.^4^ Feeding hamsters a high‐fat diet results in the development of arterial plaques in large arteries, similar to the formation of atherosclerosis plaques in humans.^5^ Extensive animal lipidomic phenotyping has demonstrated that hamsters exhibit lipid profiles resembling those of humans, such as cholesterol or triglycerides, making them an advantageous model for studying lipid disorders and related events.^6^ Hamsters synthesize apolipoprotein B‐100 (apoB‐100) in the liver and apoB‐48 in the small intestine and express CETP3, all of which align with human characteristics.^7^ Therefore, hamsters are an advantageous rodent model for studying lipid metabolism disorders associated with thrombosis.

Thromboelastography (TEG) is a widely used real‐time method for monitoring platelet function and thrombosis. It offers several advantages, including comprehensive evaluation of blood coagulation ability encompassing the coagulation cascade, platelet function, and fibrinolysis. TEG can predict the clinical efficacy of therapeutic drugs that affect coagulation ability^8^ and assess the likelihood of thrombosis, aiding in its prevention. By analyzing specific parameters, TEG can predict hypercoagulability or hypocoagulability of blood and evaluate the impact of hyperlipidemia on coagulation status, which is significant for adjusting subsequent treatment plans.^9^ Notably, in patients with coronary artery disease, TEG‐detected platelet‐fibrin clot strength is significantly correlated with major adverse cardiovascular events.^10^ TEG can also predict thrombotic risk in critically ill patients with coagulation disorders,^11^ identify and evaluate hypercoagulability in COVID‐19 patients, and predict thrombotic complications.^12^ It is a widely accepted method for detecting coagulation, thrombosis, and fibrinolysis.

However, platelets and coagulation factors are easily activated in vitro, necessitating stringent handling procedures for whole blood samples used for coagulation analysis and platelet function testing in clinical practice. Improper sample handling may lead to inaccurate results.^13^, ^14^ Therefore, experienced operators are required to monitor and analyze TEG results throughout the process.

In this study, we aimed to explore the feasibility of TEG in the detection of differences in coagulation function, platelet function, and the fibrinolytic system between hamsters and humans, providing experimental evidence for changes in blood clotting induced by dyslipidemia.

## 2 DATA AND METHODS

### 2.1 Hamsters

Fourteen male wild‐type hamsters, aged 8–10 weeks, with an average body weight of 133 ± 1.4 g, were obtained from Beijing Vital River Laboratory Animal Technology Co., Ltd. These hamsters were housed in a temperature and humidity‐controlled room with a 12/12‐h light–dark cycle and fed a normal diet. All animal experiments adhered to the current animal welfare guidelines and were approved by the Animal Care and Use Committee of Shandong First Medical University (Shandong Academy of Medical Sciences).

### 2.2 Collection of whole blood from hamsters

For whole blood collection, a total of 28 vacuum blood collection tubes with sodium citrate anticoagulation (1:9 ratio of anticoagulant to whole blood, 2‐mL capacity), 14 vacuum blood collection tubes with ethylene diamine tetraacetic acid K2 (EDTA‐K2) anticoagulation (1‐mL capacity), and 14 sets of venous blood collection needles were prepared.

Hamsters were anesthetized by intraperitoneal injection of 1% pentobarbital sodium (70 mg/kg), and their anesthesia level was monitored by toe pinching. Anesthesia was deemed appropriate when the hamsters exhibited consistent breathing patterns. The hamsters were positioned supine on foam boards, and the chest area was disinfected with alcohol. The venous blood collection needle was then inserted along the left side of the xiphoid process, close to the midline of the chest, at a 30° angle relative to the horizontal plane. Once blood return was observed at the joint between the needle and the pipeline, the other end of the blood collection needle was inserted into the vacuum blood collection tube. The needle was held firmly with the right hand, while the blood collection tube was shaken with the left hand to ensure thorough mixing of the blood and anticoagulant. The whole blood of a hamster was divided into three parts for TEG detection(2 mL), coagulation analysis (2 mL), and blood routine analysis (1 mL), respectively. After the blood collection, we used 1% pentobarbital sodium (100 mg/kg intraperitoneal injection) for euthanasia.

### 2.3 Healthy human blood sample

Fourteen healthy volunteers participated in the study, and their TEG, coagulation analysis, and blood routine results were collected. The experimental procedures were conducted under the supervision and guidance of the Ethics Review Committee of the Second Affiliated Hospital of Shandong First Medical University (ethical approval number: 2022‐162). The clinical trial registration number is ChiCTR2300077726.

### 2.4 Thromboelastography

The whole blood (2 mL) with sodium citrate anticoagulation (1:9 ratio) was collected. The KAOLIN reagent (KAOLIN, Haemonetics Corporation, batch number: HMO5628) was brought to room temperature and flicked to settle the contents at the bottom of the bottle. Then, 1 mL of blood sample was added to the reagent, allowing it to flow down along the wall. The bottle was gently inverted five times without shaking the blood sample. Next, 20 μL of 0.2 M calcium chloride was added, and 340 μL of the blood sample was drawn and added to a preheated cup (disposable cups and pins, batch number: HMO5550) in the TEG analyzer (TEG‐5000 instrument, Haemonetics Corporation). The cup tank was pushed up, and the sample was allowed to run until the maximum amplitude (MA) value was determined.

### 2.5 Coagulation analysis and blood routine

An ACLTOP750 automatic coagulation analysis assembly line (Werfen Instrument Equipment Company) was used for coagulation analysis. Prothrombin time (PT) determination kit (Instrumentation Laboratory Co, batch number: N0724173), activated partial thromboplastin time (APTT) determination kit (Instrumentation Laboratory Co, batch number: N0723779), and fibrinogen (Fib) determination kit (Instrumentation Laboratory Co, batch number: N0724166) were used. For blood routine analysis, an XN‐2000 fully automatic blood analyzer (Sysmex Shanghai Ltd.) was employed, along with the diluent for blood cell analysis (CELLPACK DCL, SYSMEX, batch number: G2433) and Fluorocell RET (SYSMEX, batch number: A2009).

### 2.6 Statistical analysis

The data were presented as mean ± standard deviation and analyzed using GraphPad Prism version 8. When the D'Agostino‐Pearson comprehensive normality test confirmed a Gaussian distribution, a *t*‐test was performed for side‐by‐side comparison. Otherwise, the Mann–Whitney test was employed.

## 3 RESULTS

### 3.1 Application of TEG to assess coagulation and platelet functions in hamsters

**FIGURE 1:**
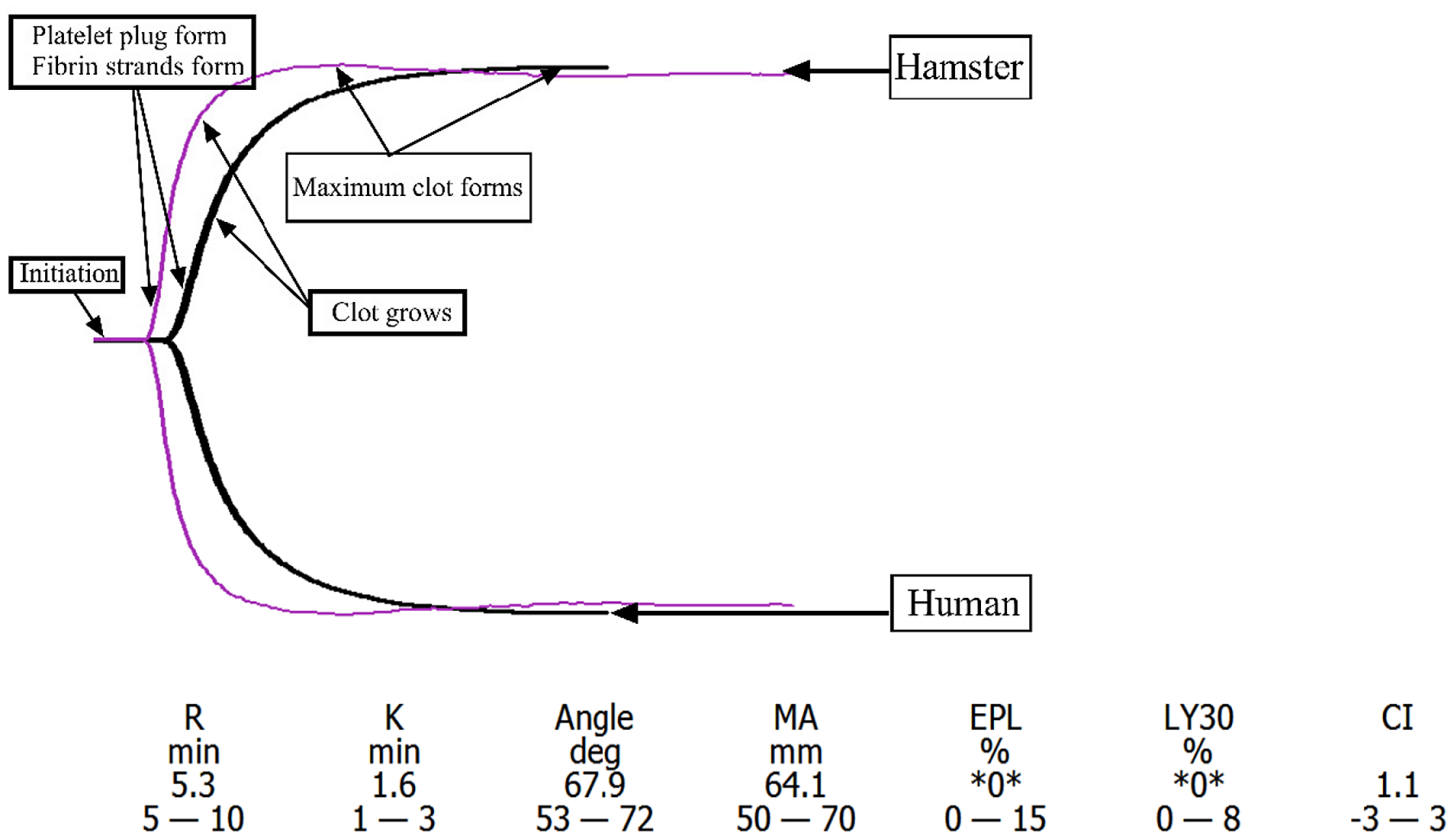
Comparison of thromboelastogram between hamsters and healthy people. The figure shows the thromboelastogram of hamsters (purple trajectory) and healthy individuals (black trajectory). Our experiments confirmed that thromboelastography (TEG) can effectively detect clotting activity and platelet function in hamsters, providing continuous monitoring and visualization of the entire coagulation process. From the figure, it is evident that hamsters have faster initial blood clot formation time and a higher rate of blood clot formation compared to humans. After the final blood clot formation, the maximum amplitude (MA), which reflects platelet function, is similar between hamsters and humans.

Commercial vacuum blood collection vessels were used to collect 6–8 mL of whole blood, which provided a convenient and smooth process that met the requirements for clinical thrombus experiments. TEG analysis of the hamster blood samples revealed a similar hemostasis process to that of humans, including coagulation initiation, platelet emboli and fibrin chain formation, blood clot increase, and blood clot lysis (Figure 1). Based on these graphical data, TEG can be employed to monitor the hemostasis, thrombosis, and platelet function in hamsters.

### 3.2 Comparison of coagulation and platelet functions between hamsters and humans

**FIGURE 2:**
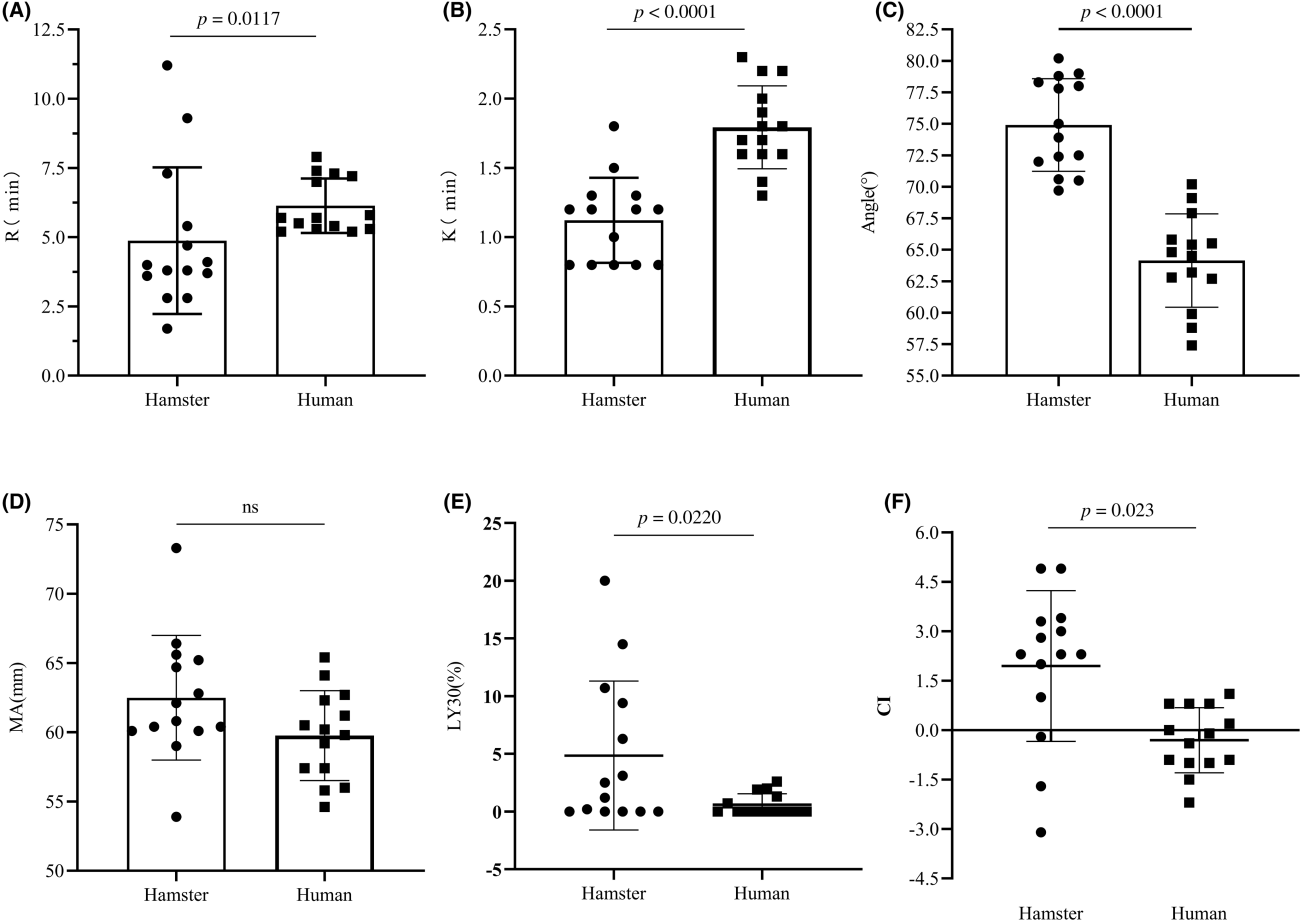
Comparison of the main parameters of thromboelastogram between hamsters and humans. This figure compares the main parameters of the thromboelastogram between hamsters and humans. It demonstrates that hamsters exhibit stronger coagulation function but similar platelet function compared to humans. The clot formation rate in hamsters is also faster than that in humans. (A) Reaction time (R value) represents the time from the start of coagulation to the formation of the first fibrin clot, reflecting the activity of coagulation factors. There are differences in R values between hamsters (*n* = 14) and healthy individuals (*n* = 14). (B) K time (K value) signifies the time required for the graph to reach 20 mm after the end point of the R time, reflecting the rate of blood clot formation and mainly indicating the function of fibrin. The K value of hamsters (*n* = 14) significantly differs from that of healthy individuals (*n* = 14). (C). The *α* angle represents the angle between the tangent line and the horizontal line of the maximum curve in the tracing diagram, reflecting the polymerization rate of the blood clot and mainly indicating the function of fibrin. The *α* angle of hamsters (*n* = 14) and healthy individuals (*n* = 14) is noticeably different. (D) Maximum amplitude (MA) is the combination of fibrin and platelets through GPIIb/IIIa receptors, representing the maximum intensity of fibrin/platelet clotting. The MA values of hamsters (*n* = 14) and healthy individuals (*n* = 14) do not show significant differences and mainly reflect platelet function. (E) LY30 indicates the amplitude attenuation rate 30 min after the maximum amplitude. There are differences in LY30 values between hamsters (*n* = 14) and healthy individuals (*n* = 14). (F) Coagulation index (CI) reflects the total clot intensity generated by all coagulation interactions and differs between hamsters (*n* = 14) and healthy individuals (*n* = 14). Data results are expressed as mean ± standard deviation (SD). Statistical analysis was performed using the Mann–Whitney test (A, B, E) and unpaired *t*‐test (C, D, F).

TEG provides key indicators such as reaction time (R value), K time (K value), alpha (*α*) angle, MA, LY30, and coagulation index (CI), etc., comprehensively reflecting the coagulation profile of the samples. The R value, reflecting the activity of blood coagulation factors, showed higher blood coagulation activity in hamster compared to healthy humans (Figure 2A). *K* value reveals the rate of blood clot formation, mainly reflecting the fibrin function. Significant differences in the *K* value (Figure 2B) indicated distinct rates of clot formation and differences in fibrin function between hamster and healthy humans. The *α* angle, which reflects the rate of blood clot polymerization, also displayed noticeable differences between hamster and healthy humans (Figure 2C). MA shows the maximum strength of fibrin and platelets to form clots, representing the platelet function. However, no significant differences were observed in the MA value (Figure 2D), indicating similar platelet functions in both species. LY30 reflects variations in hemolysis and fibrinolysis. It demonstrated differences between hamster and healthy humans (Figure 2E). CI represents total clot strength produced by all coagulation interactions. There were differences in the CI values between hamster and healthy humans (Figure 2F), indicating that the overall coagulation function of hamster was higher than hypercoagulability observed in humans.

### 3.3 Fast clotting time and low fibrin content of hamsters

**FIGURE 3:**
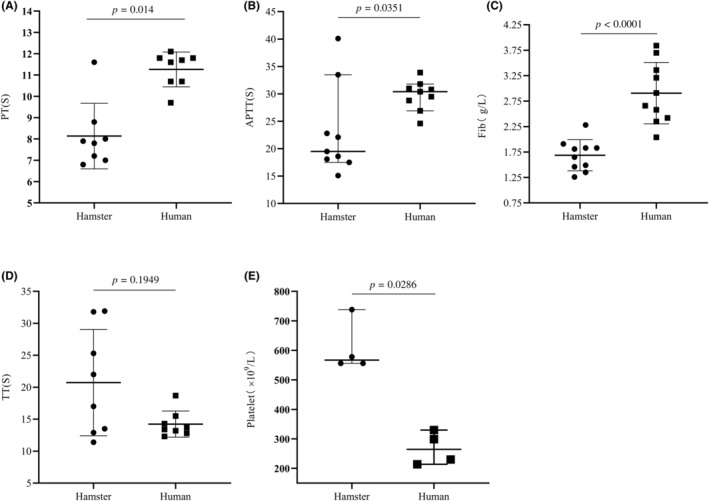
Comparison of coagulation time and fibrin content between hamsters and humans. This figure demonstrates that hamsters have a faster coagulation time and significantly lower fibrinogen (Fib) concentration compared to humans. The ACLTOP750 automatic coagulation analysis line was used to detect coagulation routine parameters, including Fib, prothrombin time (PT), activated partial thromboplastin time (APTT), Thrombin time (TT), and platelet count. (A) PT shows differences between hamsters (*n* = 8) and healthy individuals (*n* = 8), indicating higher quantity and function of thrombin factors involved in the exogenous thrombin pathway in hamsters compared to healthy individuals. (B) APTT results indicate differences in APTT between hamsters (*n* = 9) and healthy individuals (*n* = 9), suggesting a higher number or function of coagulation factors involved in the endogenous coagulation pathway of hamsters compared to healthy individuals. (C) The results show that there is a significant difference in Fib concentration between hamsters (*n* = 10) and healthy individuals (*n* = 10). (D) TT results show no significant difference between hamsters (*n* = 8) and healthy individuals (*n* = 8). (E) The XN‐2000 automatic blood analyzer was used to determine platelet count, which shows a difference between hamsters (*n* = 4) and healthy individuals (*n* = 4). Data are expressed as mean ± standard deviation (SD). Statistical analysis was performed using the Mann–Whitney test (A, D) and unpaired *t*‐test (B, C, E).

To validate the reliability of the hamster blood collection process, routine blood tests were conducted on hamster samples. The results confirmed the consistency of red blood cell count, white blood cell count, hemoglobin content, and hematocrit with previous reports^15^ (Table 1), supporting the reliability of the blood collection method using clinical vacuum blood collection vessels for hamsters. Although the platelet counts in hamster exceeded that of healthy humans (Figure 3E), their platelet aggregation function did not show a significant increase (Figure 2D). Regarding coagulation routine analysis, the Fib content in hamster was notably lower compared to healthy humans (Figure 3C). However, intriguingly, TEG results revealed robust functionality of Fib in hamster.

**TABLE 1:** Result of Hamster blood routine.

PLT (10^3^/μL)	RBC (10^6^/μL)	HGB (g/dL)	HCT	WBC (10^3^/μL)	MCV (fl)
607 ± 87.95	8.843 ± 0.58	16.8 ± 3.86	0.48 ± 0.01	7.578 ± 1.49	54.45 ± 4.32

Abbreviations: HCT, hematocrit; HGB, hemoglobin; MCV, mean cell volume; PLT, platelets; RBC, red blood cells; WBC, white blood cells.

PT, a screening test for the function of the exogenous coagulation system, exhibited significant differences between hamster and healthy humans (Figure 3A). The PT value was lower than that of healthy humans, suggesting that the number or function of coagulation factors involved in the exogenous coagulation pathway of hamster was larger or stronger than that of healthy humans. The APTT is a widely employed screening tool for detecting defects in coagulation factors (VII, IX, and XII) within the endogenous pathway. No significant differences in APTT between hamster and healthy humans were observed (Figure 3B), indicating similarity in terms of the number or function of coagulation factors involved in the endogenous coagulation pathway. Thrombin time (TT) refers to the time of blood coagulation after standardized thrombin is added into plasma, reflecting the conversion of Fib to fibrin. However, there were no significant differences in TT between hamster and healthy humans (Figure 3D), suggesting a similar common coagulation pathway. In summary, the results of coagulation routine analysis indicated that the exogenous and endogenous coagulation times in Syrian hamster were faster than those in healthy humans, consistent with the TEG findings. Moreover, the content of Fib was significantly lower than that of healthy humans (Figure 3C), whereas the time for the conversion of Fib into fibrin was similar.

## 4 DISCUSSION

The hamster is a widely used rodent in various experimental models, including oncology, immunology, physiology, and reproductive biology.^16^ It shares many similarities with humans in terms of lipid metabolism characteristics, such as lipoprotein synthesis, processing, ligand binding, and recycling.^18^ Due to its convenience for surgical operations, blood collection, and tissue sampling, hamsters are frequently used to study vascular diseases associated with lipid metabolism disorders. Moreover, hamsters are convenient for surgical operation, blood collection, and tissue sampling. The amount of blood collected from one hamster is enough for TEG, coagulation routine, and blood routine at the same time. In this comparative study, we investigated thrombosis functions using TEG and found that hamsters had platelet function similar to humans, but their coagulation function was significantly stronger.

TEG has gained popularity as an instrument for clinical hemostasis and thrombosis detection due to its advantages over traditional coagulation analyses. It is designed based on the current basic coagulation model, emphasizing the crucial role of platelets. In our study, TEG successfully detected thrombus formation and platelet function in hamsters.

To ensure proper sampling, we conducted a blood routine test on hamsters using the XN‐2000 fully automatic hematology analyzer. The use of a commercial vacuum blood sampling kit proved applicable to hamsters, particularly in conjunction with automatic detecting instruments. As hamsters have a larger average weight, surgical operations, tissue and blood collection are more convenient. Vacuum blood collection kits, along with human testing equipment, can be used without the need for specific animal equipment.

Analysis of whole blood samples from hamster using TEG revealed a hemostasis process consisting of initiation, amplification, and magnification, similar to humans.^17^ Compared to healthy individuals, hamster exhibited higher activity of coagulation factors and hypercoagulability, as indicated by the CI value. Platelet function, which plays a key role in arterial thrombosis, did not show significant differences between hamster and humans, suggesting the suitability of hamsters for studying platelet‐dominated arterial thrombosis.

We also conducted the ACLTOP750 automatic coagulation analysis pipeline to confirm that the initiation of coagulation in hamsters was similar to that in humans, consistent with the TEG results. The PT of exogenous coagulation time was significantly shorter in hamster compared to healthy humans, suggesting differences in coagulation factors involved in the coagulation pathway. However, no significant differences were observed in APTT, indicating similarity in the endogenous coagulation pathway between hamster and humans. TT revealed a comparable transformation time from Fib to fibrin in hamster and humans, suggesting that Fib function might have little relation to its content.

The TEG results for K value and *α* angle indicated stronger fibrin function in hamster compared to humans, despite lower Fib content observed through coagulation routine analysis. The low Fib content may be attributed to the significantly higher platelet count in hamster, leading to enhanced interaction between fibrin and platelets and rapid clot formation, explaining the strong Fib function observed in TEG. MA, reflecting the maximum strength of the fibrin/platelet clot, was mainly influenced by Fib and platelets, with platelets demonstrating a greater effect. Platelet aggregation function, expressed by the MA value, did not exhibit significant differences between hamster and healthy humans. Although hamster had diminished Fib levels, it did not impede blood clot formation. However, rapid clot dissolution in hamster, as indicated by the LY30 value, suggested heightened fibrinolysis potentially due to the lower Fib content.

Although we found that the application of TEG can comprehensively assess the risk of thrombosis and bleeding in hamsters, due to the hypercoagulable state of hamsters, there are certain limitations in using TEG to study the coagulation status after trauma and massive bleeding. Therefore, rodents are used for research in coagulation function and signaling pathways due to the convenience in genetic modification.^18^, ^19^, ^20^, ^21^ The disadvantage of mice is small total blood volume. When performing TEG detection, the reaction system needs to be reduced based on the proportion,^21^ or more samples are pooled together for detection.^19^ Therefore we conducted this study to compare the TEG parameters between human and hamster.

Our findings suggest the importance of establishing internal reference ranges for specific TEG parameters in hamsters to improve the validity and reliability of analysis when using hamsters as animal models.^22^

## 5 CONCLUSION

In summary, the use of whole blood samples from hamsters enables a comprehensive evaluation of thrombosis and bleeding risks. The process of hemostasis and thrombosis in hamsters closely resembles that in humans, making them suitable models for studying these phenomena. Hamsters offer advantages in terms of reduced equipment costs, the number of animals required, and operational convenience.

## AUTHOR CONTRIBUTIONS

Yu Yang conceived and designed this study; Ze Yang and Jingjing Ba took charge of animal experiments; Lili Xie was responsible for the clinical data collection; Simin Zan, Letong Zhang, and Xinyi Zhang handled the animal breeding and experiments; Yang Ze, Lili Xie, and Jingjing Ba made equal contributions to this study. Yang Ze and Yu Yang drafted the manuscript, Yu Yang and Yang Ze conducted the data analysis. Yu Yang was responsible for funding. All authors read and approved the manuscript.

## FUNDING INFORMATION

This research is supported by the general program of National Natural Science Foundation of China (81970385), Shandong Natural Science Foundation (ZR2019MH021), and Student Research Training Program (2022104391282).

## CONFLICT OF INTEREST STATEMENT

The authors declared no competing interests.

## ETHICS STATEMENT

All hamsters were housed in a temperature and humidity‐controlled room with a 12/12‐h light–dark cycle. All animal experiments were approved by the Animal Care and Use Committee of Shandong First Medical University (Shandong Academy of Medical Sciences) and conformed to the current animal welfare guidelines. TEG, coagulation analysis, and blood routine results of eight healthy volunteers were collected, and relevant experimental procedures were carried out under the supervision and guidance of the Ethics Review Committee of the Second Affiliated Hospital of Shandong First Medical University. (The ethical approval number is 2022‐162). The registration number of clinical trial is ChiCTR2300077726.
